# Regulatory helix plays a key role in genetic ON–OFF switching for the 2′-deoxyguanosine-sensing mRNA element

**DOI:** 10.1016/j.jbc.2025.110282

**Published:** 2025-05-22

**Authors:** Susmit Narayan Chaudhury, Nathan Edward Jespersen, Scott P. Hennelly, Karissa Y. Sanbonmatsu

**Affiliations:** 1 Theoretical Biology and Biophysics, Los Alamos National Laboratory, Los Alamos, New Mexico, USA; 2 Genomics and Bioanalytics Group, Biosciences Division, Los Alamos National Laboratory, Los Alamos, New Mexico, USA; 3 New Mexico Consortium, Los Alamos, New Mexico, USA

**Keywords:** RNA structure, RNA biochemistry, RNA folding, riboswitch

## Abstract

Transcriptional riboswitches, noncoding mRNA elements that operate in *cis* to regulate gene expression, have promising potential in medicine, synthetic biology, and directed evolution. They bind to cellular metabolites or metal ions with high specificity, leading to conformational rearrangements that facilitate the activation or premature termination of transcription for downstream genes. This elegant mechanism for feedback regulation of metabolic pathways has been identified in prokaryotes and a few eukaryotes. Our chemical probing of the 2′-deoxyguanosine (2′-dG)-sensing riboswitch demonstrates that the overall conformational state of the full-length riboswitch (dGsw-fl) is unresponsive to the 2′-dG. Although binding proceeds as expected, dGsw-fl exclusively populates an OFF state of transcriptional inhibition. We chemically probed the structure of a known dGsw transcriptional intermediate (dGsw-int) to evaluate the possibility of a cotranscriptional regulatory role. Interestingly, apo dGsw-int adopts an alternative conformation in which a stable anti-terminator helix is formed, leading to an ON state where transcription can proceed. In the presence of 2′-dG, this anti-terminator helix is destabilized to produce a conformation reminiscent of the full-length, OFF-state dGsw. Using a fluorescence quenching assay, we demonstrate that binding 2′-dG to early transcriptional intermediates can inhibit the formation of the anti-terminator helix, locking dGsw in an OFF state. These data suggest that metabolite sensing occurs during a brief window of time between the synthesis of two transcriptional intermediates. Our studies indicate that dGsw does not function as a binary ON−OFF switch but instead fine-tunes the transcription of downstream genes during RNA synthesis using key intermediates.

Riboswitches are broadly distributed throughout prokaryotic genomes, where they act as sensors that respond to concentrations of essential small molecules such as signaling molecules, nucleotides, vitamins, metal ions, and amino acids (1, 2, 3, 4, 5, 6, 7, 8, 9, 10, 11, 12). These RNA-based sensors typically comprise a sensory, or “aptamer” domain, and a regulatory domain (Fig. 1*A*). The aptamer serves as a highly specific receptor for particular metabolites and is often well-conserved in sequence and structure. In most cases, the regulatory domain overlaps with the expression platform, and the conformation adopted by the aptamer-regulatory domain pair determines the expression level of downstream genes. Metabolite binding to the aptamer domain of transcriptional riboswitches leads to structural rearrangements that favor either premature termination of transcription (“OFF switch”) or continuation of transcription (“ON switch”) (13, 14, 15, 16).

**Figure 1:**
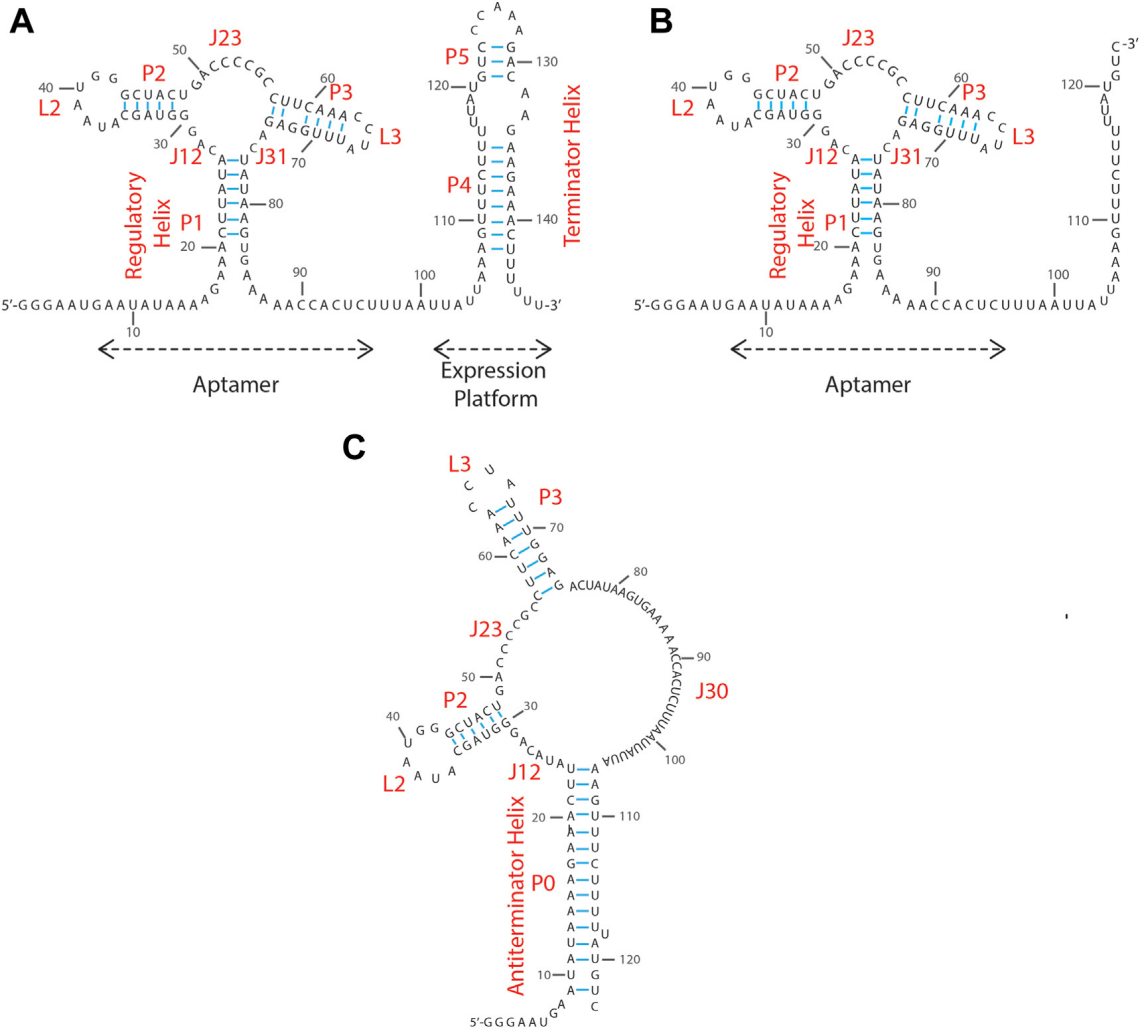
**Sequence and predicted secondary structure of dGsw constructs**. *A*, full-length transcript, (*B*) an intermediate transcript OFF state, and (*C*) ON state of intermediate transcript of dGsw. For the intermediate transcript, the 3′ complementary segments of P4 and P5 helices are trimmed off to eliminate the possibility of forming a terminator helix. The structural elements (helices, junctions, and loops) are labeled in *red*.

As cotranscriptional riboswitches operate during transcription, metabolites must bind to the aptamers before the RNA polymerase reaches the termination point to affect expression levels. Riboswitch regulation, therefore, resembles a race between ligand binding and RNA polymerase extension. For “OFF state” regulators, transcription is terminated if ligands bind before the termination point is reached but continues if ligands bind afterward. Intriguingly, studies on the FMN-sensing riboswitch have demonstrated that premature termination requires a concentration of metabolite substantially higher than the K_D_, as termination is governed by the kinetics of ligand binding rather than affinity in this system (17). In contrasting cases, such as within the purine riboswitch family, RNA polymerase pauses during riboswitch transcription, providing additional time for ligand-binding (18).

The purine riboswitch family regulates genes responsible for purine biosynthesis and transport in prokaryotes (4, 5, 9). Members of this family adopt a highly conserved “tuning fork” structure within their aptamer domain (Fig. 1*A*), and display high sequence conservation (4, 5, 9). Despite their structural similarities, purine riboswitches exhibit marked regulatory diversity. For example, the *xpt-pbuX* riboswitch from *Bacillus subtilis* controls the genetic regulation of xanthine phosphoribosyltransferase and purine permease genes (4) and operates as a transcriptional OFF switch (4). In contrast, the *B*. *subtilis pbuE* riboswitch regulates purine efflux pump genes as a transcriptional ON switch (9, 19), where binding of a ligand enables transcription of downstream genes. These results demonstrate that riboswitches display remarkable regulatory diversity and combine variable on-off switching mechanisms with the complex kinetic controls described earlier.

The inherent variability in riboswitch functionality necessitates specific studies detailing the regulatory mechanisms employed by each riboswitch. Here, we describe *Mesoplasma florum* 2′-dG riboswitch (dGsw) regulation of the downstream ribonucleotide reductase gene (5, 20). To elucidate the precise relationship between structural rearrangements and gene regulation, we used selective 2′-hydroxyl acylation analyzed by primer extension (SHAPE) (21). SHAPE is a chemical probing-based method useful for evaluating local backbone flexibility of RNA molecules. Briefly, flexible nucleotides adopt local conformations that increase the nucleophilic reactivity of 2′-hydroxyl groups towards electrophilic reagents, such as 1-Methyl-7-nitroisatoic anhydride (1M7). More structured or rigid nucleotides infrequently adopt these conformations. Therefore, high SHAPE reactivity values serve as a descriptive proxy for locally unstructured nucleotides.

In this study, SHAPE is utilized to determine the secondary structure of the dGsw full-length (dGsw-fl, Figu. 1*A*) transcript and one of the transcriptional intermediates (dGsw-int; Fig. 1, *B* and C) in both ligand-bound and ligand-free states. Our data suggest that a transcriptional pause site allows dGsw to adopt two highly distinct conformations (Fig. 1, *B* and C). In the ligand-free state, dGsw-int favors a conformation with weaker association within the P1 helix, promoting the formation of an anti-terminator helix (AT-helix; Fig. 1*C*). This anti-terminator conformation turns ON the downstream gene. In the presence of ligand, the anti-terminator helix is disfavored. This facilitates the formation of a terminator helix and leads to premature termination of transcription. These findings are supported by a “two piece” kinetic assay with 2-aminopurine (2-AP) (22), which we used to investigate how the ON and OFF states of dGsw depend on the formation-dissociation equilibrium of helix P1. The assay shows that the anti-terminator strand is in competition with 2′-dG for binding near helix P1. Our results suggest that cotranscriptional folding into a trapped conformation green-lights transcription, but that polymerase pausing provides a brief window for riboswitch remodeling to facilitate ligand-dependent gene repression.

## Results

### Conformational analysis of dGsw RNA constructs

To investigate the structural properties of the dGsw RNA constructs, we first examined their conformational homogeneity using 8% native TBM polyacrylamide gel electrophoresis (PAGE) (Supplementary Method 1 and Fig. S3) in the absence and presence of 2′-dG. The PAGE revealed the presence of a single predominant conformation for both dGsw-fl and dGsw-int RNA constructs, regardless of 2′-dG presence. This observation suggests that the RNAs, under the specified solution conditions, primarily adopt a single overall structure, and that the presence or absence of the ligand does not induce large-scale conformational changes that would be detectable by gel electrophoresis.

### Ligand-induced structural collapse is confined to the aptamer domain of dGsw

**Figure 2:**
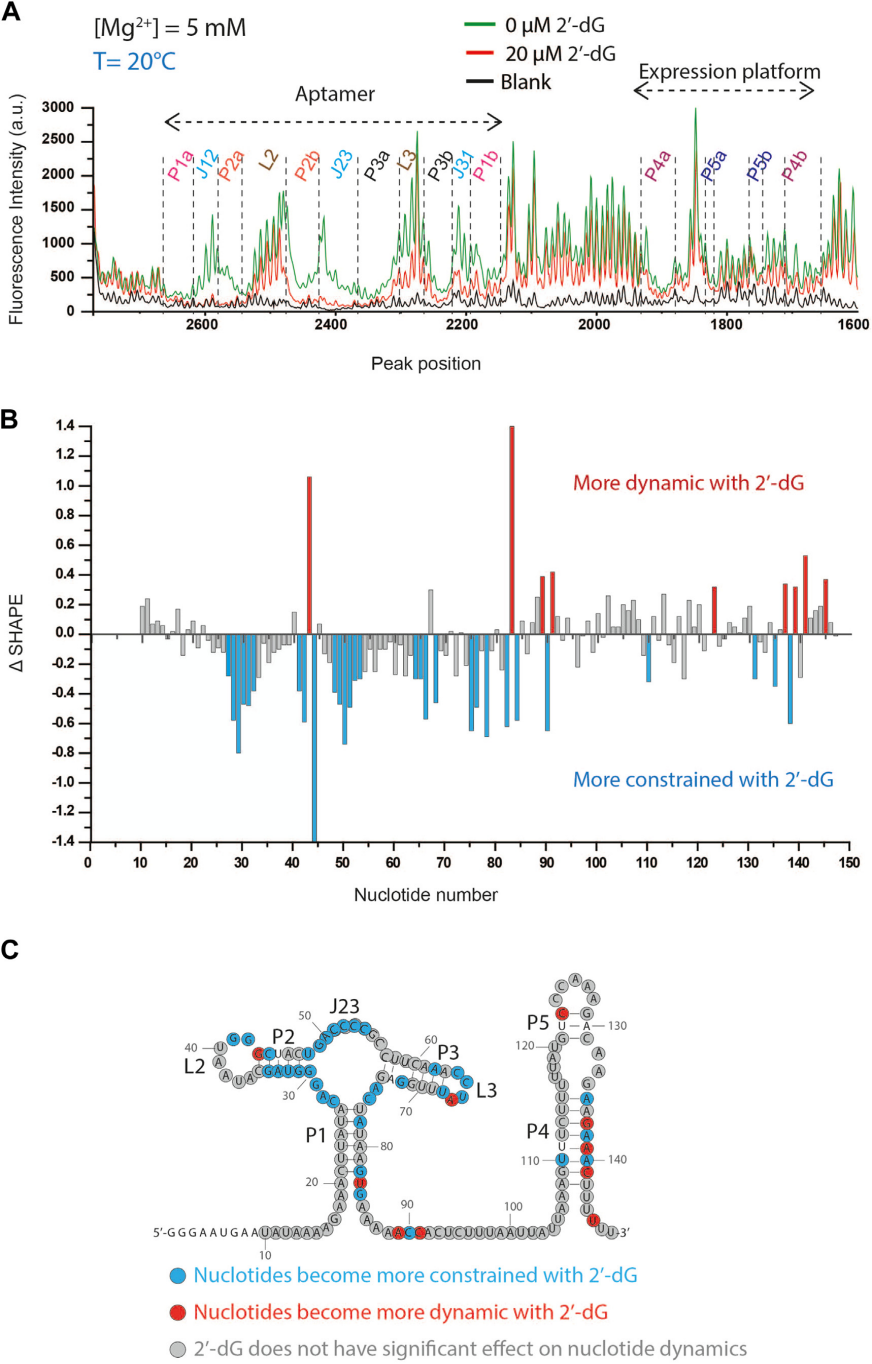
**Secondary structure of the expression platform of the full-length transcript of dGsw (dGsw-fl) is independent of ligand**. *A*, representative electropherogram in presence of 5 mM of Mg^2+^. The overlaid traces depict the data without 2′-dG (*green*) and with 20 μM 2′-dG (*red*). The nucleotides from the different structural regions analyzed here are indicated. A static electropherogram at the expression platform has indicated that base-pairing interactions remain unaltered in response to the addition of the ligand. The SHAPE reactivities as a function of nucleotide position are evaluated by subtracting the integrated area for individual nucleotides corresponding to the blank sample (RNA treated with neat DMSO) from SHAPE data (1M7 treated). To normalize the areas, the first 10% of the data corresponding to the highest reactivity values are considered outliers and are temporarily excluded from the analysis. From the remaining data, 10% of highly reactive nucleotides are averaged to calculate a normalization factor. The entire profile, including the previously excluded outliers, is then normalized by that factor. *B*, difference plot (Δ SHAPE) showing changes in nucleotide flexibility upon addition of 2′-dG. Positive values (*red bars*) indicate nucleotides that become more dynamic with 2′-dG, while negative values (*cyan bars*) represent nucleotides that become more constrained with 2′-dG. *Gray bars* indicate positions where 2′-dG has minimal effect on nucleotide dynamics. *C*, secondary structure model of the 147-nucleotide dGsw-fl RNA with nucleotides color-coded according to their response to 2′-dG binding. *Red* circles indicate nucleotides that become more dynamic in the presence of 2′-dG, *cyan* circles show nucleotides that become more constrained, and *gray* circles represent nucleotides where the ligand has minimal effect on dynamics. Nucleotide positions are numbered, and structural elements (P1-P5, L2, L3, J23) are labeled. Unmarked nucleotides were unanalyzable in the SHAPE experiment.

To gain insights into the structural rearrangements of dGsw-fl upon ligand binding, we used SHAPE probing in the absence and presence of 2′-dG. Although the general conformation is conserved between the two states, ligand interactions trigger a collapse of the aptamer domain by stabilizing the interhelical junctions (Fig. 2), encapsulating 2′-dG. These findings are corroborated by previous structural studies of the ligand-bound aptamer (23, 24, 25), which show that A26, C27, C52, C53, C54, G55, and C76 directly interact with or stabilize 2′-dG in the binding pocket (26, 27). SHAPE data demonstrate that regions surrounding these nucleotides are considerably stabilized by 2′-dG interactions (Fig. 2). In contrast, a majority of the P2 and P3 helices, apart from the G30-U48 pair, are become structured upon ligand addition (Fig. 2, *B* and C), in agreement with the previous NMR experiments (28). Notably, the terminal G30-U48 pair becomes structured in the simultaneous presence of Mg^2+^ and 2′-dG (Fig. 2, *B* and C and Fig. S2). These observations indicate that conformational rearrangements by 2′-dG are limited to local restructuring rather than global refolding.

The SHAPE experiments reveal consistently low reactivity in the 5′ complementary region of the P1 helix (C21-A26) (Fig. 2). This observation suggests that the structural integrity of the C21-A26 segment remains unchanged, irrespective of the presence of 2′-dG. SHAPE reactivity of the 3′-complementary regions of the P1 helix (P1b; U77 to G82) is reduced in the presence of a ligand (Fig. 2, *B* and C), suggesting the P1 helix is stabilized in the ligand-bound state. This conclusion is consistent with the results from single-molecule force spectroscopy on a related purine riboswitch, where the P1 helix of an adenine riboswitch is dissociated in the apo state but folds after adenine binding (29).

Analysis of the electropherogram for the regulatory domain region (nucleotides 90–143) revealed identical conformations for both the apo and holo states (Fig. 2, *A*–C). Therefore, the terminator helix (P4 and P5) is formed independent of 2′-dG. This conformation is consistent with the functional OFF-state, signifying that the dGsw-fl RNA cannot adopt an ON-state fold once fully formed. Interestingly, we still observe a structured terminator helix without Mg^2+^, suggesting it is intrinsically stable (Fig. S2).

**Figure 3:**
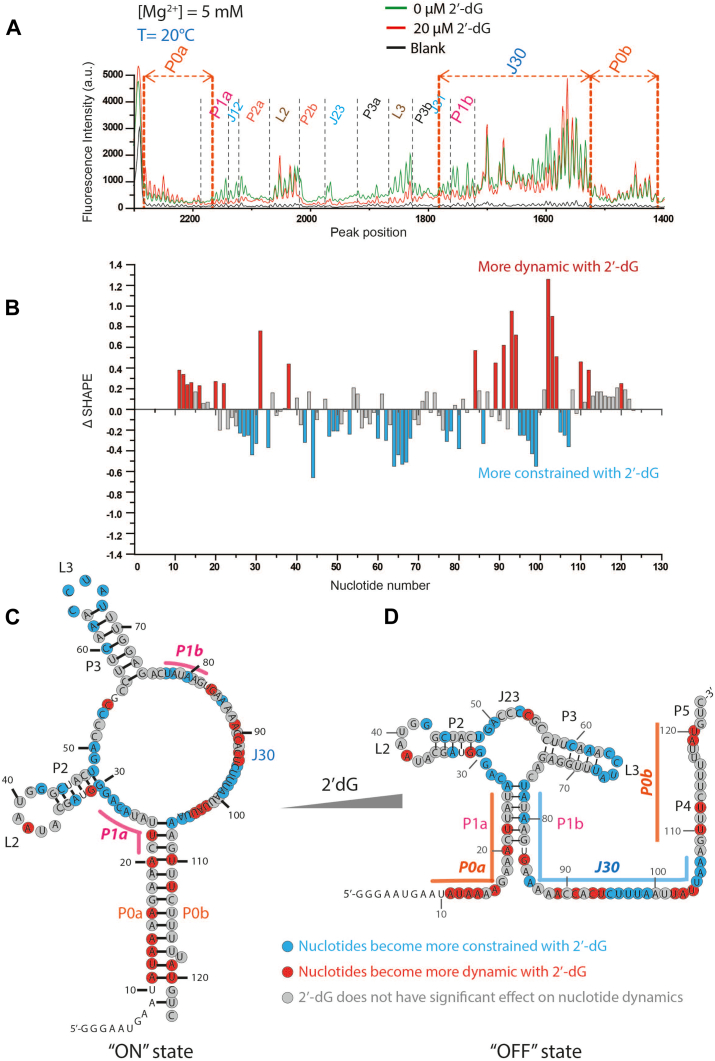
**dGsw-int occupies alternative conformations based on occupancy state**. *A*, representative electropherogram of dGsw-int RNA in the presence of 5 mM of Mg^2+^. Overlaid traces depict the data without 2′-dG (*green*) and with 20 μM 2′-dG (*red*). The nucleotides from the different structural regions analyzed here are indicated. The SHAPE reactivities as a function of nucleotide position are evaluated by subtracting the integrated area for individual nucleotides corresponding to the blank sample (RNA treated with neat DMSO) from SHAPE data (1M7 treated). To normalize the areas, the first 10% of the data corresponding to the highest reactivity values are considered outliers and are temporarily excluded from the analysis. From the remaining data (areas), 10% of highly reactive nucleotides are averaged to calculate a normalization factor. The entire profile, including the previously excluded outliers, is then normalized by that factor. The normalized SHAPE reactivities for (*B*) difference plot (Δ SHAPE) showing changes in nucleotide flexibility upon addition of 2′-dG. Positive values (*red bars*) indicate nucleotides that become more dynamic with 2′-dG, while negative values (*cyan bars*) represent nucleotides that become more constrained with 2′-dG. *Gray bars* indicate positions where 2′-dG has minimal effect on nucleotide dynamics. SHAPE derived secondary structure model of the 123-nucleotide dGsw-int RNA in (*C*) “ON” (apo) state and (*D*) “OFF” (holo) state, with nucleotides color-coded according to their response to 2′-dG binding. *Red* circles indicate nucleotides that become more dynamic in the presence of 2′-dG, *cyan* circles show nucleotides that become more constrained, and *gray* circles represent nucleotides where the ligand has minimal effect on dynamics. Nucleotide positions are numbered, and structural elements (P1-P5, L2, L3, J23) are labeled. Unmarked nucleotides were unanalyzable in the SHAPE experiment.

With the full-length construct locked into the OFF-state, we set out to determine whether stable transcriptional intermediates are required to produce the ON-state structure. Previous work has demonstrated that RNA polymerase pauses for an extended time at two sites in dGsw: around nucleotides 93 to 103 for an average of 7 s and again near nucleotide U118 for approximately 11 s (30). We therefore designed constructs encompassing either the first 123 nucleotides (dGsw-int) or the first 98 nucleotides making up the aptamer domain (dGsw-apt). In our previous publication, we demonstrated that the 98-nucleotide-long dGsw-apt construct fully formed the aptamer domain (P1_closed_ conformation) in a cumulative response to 2′-dG and Mg^2+^ (31). In the dGsw-int construct, the 3′ end of P4 and P5 helices are deleted, eliminating the possibility of forming a terminator helix (Figs. 1*B* and 3).

SHAPE experiments on dGsw-int reveal a collapsed conformation of the aptamer domain in the presence of 2′-dG, consistent with what is seen in the full-length structure (Figs. 2*C*, 3*C*). As expected, the 3′ flanking region (nts 83–123) is moderately to highly SHAPE reactive, indicating these nucleotides are largely unstructured (Fig. 3, *A* and C). Interestingly, analysis of dGsw-int in the absence of ligand revealed a completely different SHAPE electropherogram (Fig. S3, *A*, *B* and *D*). The 5′-segment of dGsw-int RNA (nts 11–23) shows very little SHAPE reactivity in the apo state (Fig. 3, *A*, *B* and *D*), with normalized values markedly lower than those of the holo state (Fig. 3*C*). These observations suggest the involvement of nucleotides 11 to 23 in the formation of a structured region. The SHAPE reactivities of the subsequent nucleotides (24, 25, 26, 27, 28, 29) are increased in the apo state, indicating the P1 helix is more flexible in the absence of ligand. Helices P2, P3, and J23 display lower reactivities in the holo state, consistent with collapse of the aptamer domain in the presence of 2′dG (Fig. 3, *A*, *B* and *D*). After the P3 helix, a long-disordered segment (nts 75–106) dotted with a patch of two well-ordered residues (C93, U94) is observed (Fig. 3, *A*, *B* and *D*). Finally, residues 107 to 121 display significant attenuation in SHAPE reactivities in the apo state, evincing the presence of an ordered segment closing the anti-terminator helix (AT) (Fig. 3*A*).

Previous NMR spectroscopy studies identified a genetically ON state with distinct major and minor subpopulations (28). Our SHAPE data corroborate these findings, and the secondary structure identified here for apo dGsw-int is consistent with an ON-state conformation. Interestingly, the minor subpopulation identified *via* NMR harbored an interior loop within an elongated anti-terminator helix (28). The low SHAPE reactivity for residues C93 and U94 indicates that our SHAPE data capture both the major and minor ON-state conformations.

### Validation of SHAPE analysis and structural ensemble analysis

To ensure the robustness of our SHAPE data analysis and to gain deeper insights into the structural ensembles of the riboswitch, we employed two independent computational methods: RNAProbe (32) and RNAstructure (33).

SHAPE reactivity data provides a nucleotide-resolution snapshot of RNA flexibility. High SHAPE reactivity indicates that a nucleotide is relatively unconstrained and, therefore, likely to be unpaired. In contrast, low reactivity suggests that a nucleotide is involved in base pairing or is otherwise structurally constrained. RNAProbe analysis (32) corroborated the findings obtained using our in-house scripts, which we have successfully implemented to analyze the SHAPE-derived secondary structure of various RNAs in our earlier works (22, 34, 35, 36, 37, 38, 39), confirming the reliability of our methodology and conclusions. Most importantly, the regions of high and low reactivity predicted by RNAProbe were consistent with the unpaired and paired nucleotides in our SHAPE-derived structural models. This agreement between the two analysis methods increases our confidence in the accuracy of the SHAPE-derived structural information.

To further investigate the potential for structural ensembles and to explore the range of conformations consistent with our SHAPE data, we utilized the RNAstructure tool (33). This analysis was conducted for all four experimental conditions: dGsw-fl + 2′-dG (Fig. S5), dGsw-fl (Fig. S6), dGsw-int + 2′-dG (Fig. S7), and dGsw-int (Fig. S8). The RNAstructure algorithm generates multiple potential RNA secondary structures and their associated thermodynamic free energies. By incorporating our SHAPE reactivity data as constraints, we could refine the structural predictions and identify the most probable conformations (Subpanel A in each Fig. S5–8). This approach allowed us to assess the degree of structural heterogeneity in our samples. This analysis generated multiple potential structural conformations for each construct. Notably, the lowest-energy structures within each condition shared core structural features, with only minor variations in peripheral regions. This analysis supports our conclusion regarding the absence of dramatically different structural conformations, instead showing variations in local structure rather than fundamentally distinct global folds. The energy differences between conformations within each condition are relatively small, suggesting that the RNA likely samples a continuum of similar structures around a dominant conformation rather than exhibiting distinct structural states separated by significant energy barriers.

### Formation of the anti-terminator helix is 30 times faster in the absence of ligand

**Figure 4:**
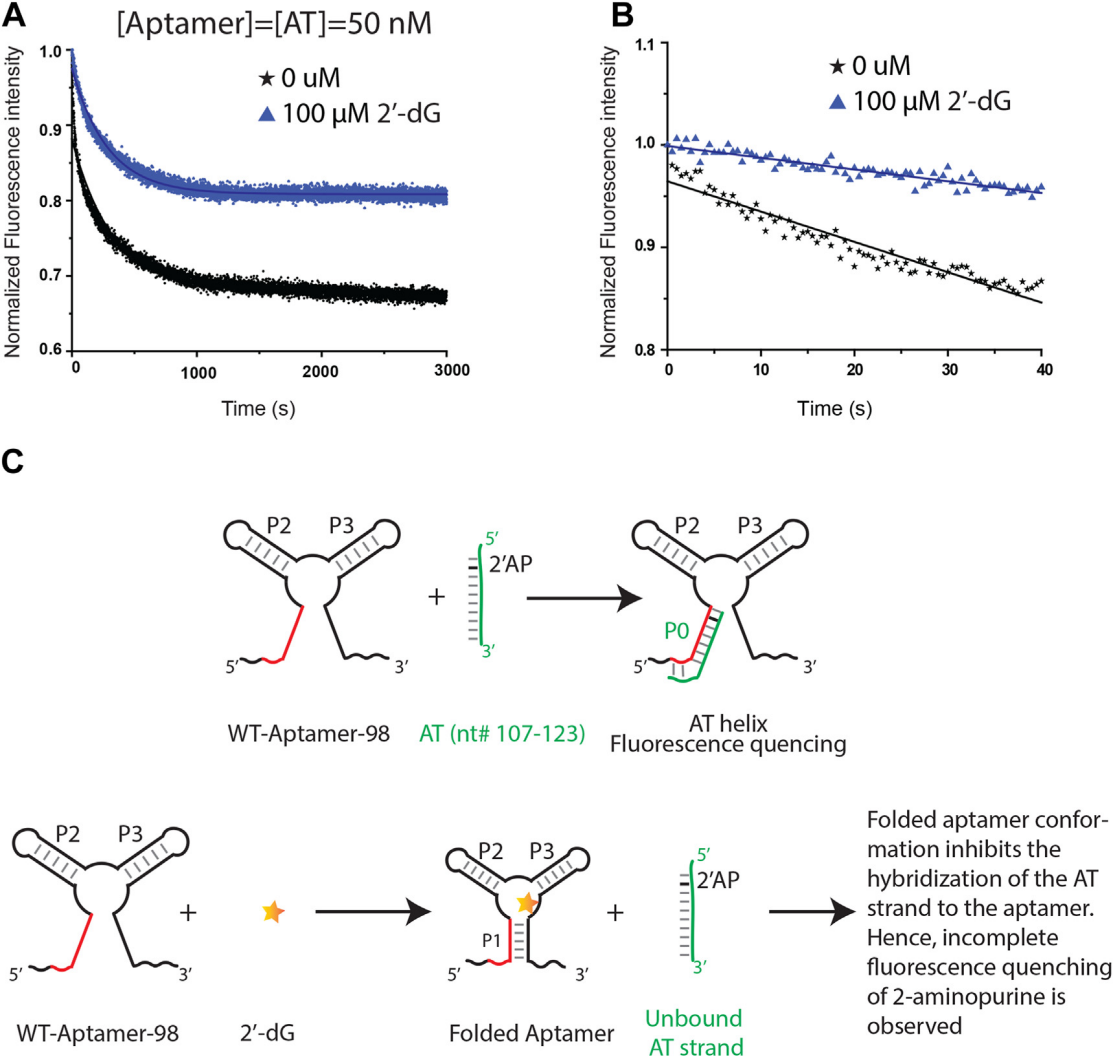
**Formation of the anti-terminator strand is 25 times faster in the absence of ligand**. *A*, 50 nM of each aptamer RNA and 2-aminopurine labeled AT strand are rapidly mixed in the absence and presence of 100 μM of 2′-dG (final concentration). *B*, the observed rate constants (k_obs_) in the presence or absence of ligands were calculated by fitting initial 40s of kinetic data in a single exponential decay function. *C*, scheme for the kinetic switching process. In the ligand-free state 2-Aminopurine-labelled AT strand anneals with the aptamer domain, forming the AT helix and quenching 2-AP fluorescence. The P1 helix closure is triggered by 2′-dG, which inhibits hybridization.

To understand the kinetics of the formation of the anti-terminator helix, we developed a two-piece assay to measure binding interactions between dGsw-apt and an AT-strand labeled with 2-aminopurine (2-AP; Fig. 4*A*). 2-AP is an adenine analog whose fluorescence is sensitive to the environment and has been successfully used to report on structural changes in other riboswitches (22, 40). Within the AT-strand (nts 107–123), A108 is substituted by 2-AP (Fig. 4*A*). First, we studied the association of the AT strand to the aptamer domain by monitoring the quenching of 2-AP fluorescence for 3000 s in the absence of ligand (Fig. 4*B*). The k_obs_ for interactions between apo dGsw-apt and the AT-strand are 2.9 × 10^-3^± (1.00 × 10^-4^) s^-1^. In contrast, when dGsw-apt is preincubated with excess 2′-dG, the rate of 2-AP fluorescence quenching increased by an order of magnitude (1.0 × 10^-2^ ± (5.07 × 10^-5^)) s^-1^. These findings strongly suggest that, in the presence of excess ligand, the 5′ complementary nucleotides (nts 9–23) of the anti-terminator helix are protected and less likely to hybridize with the AT strand. This conclusion is supported by our SHAPE results for apo dGsw-int (Fig. 3, *B* and *D*), which revealed that the regulatory helix P1 is dissociated in the absence of 2′dG (Fig. 3*B*), consistent with the dissociation of these residues to enable the rapid association of the AT-helix.

**Figure 5:**
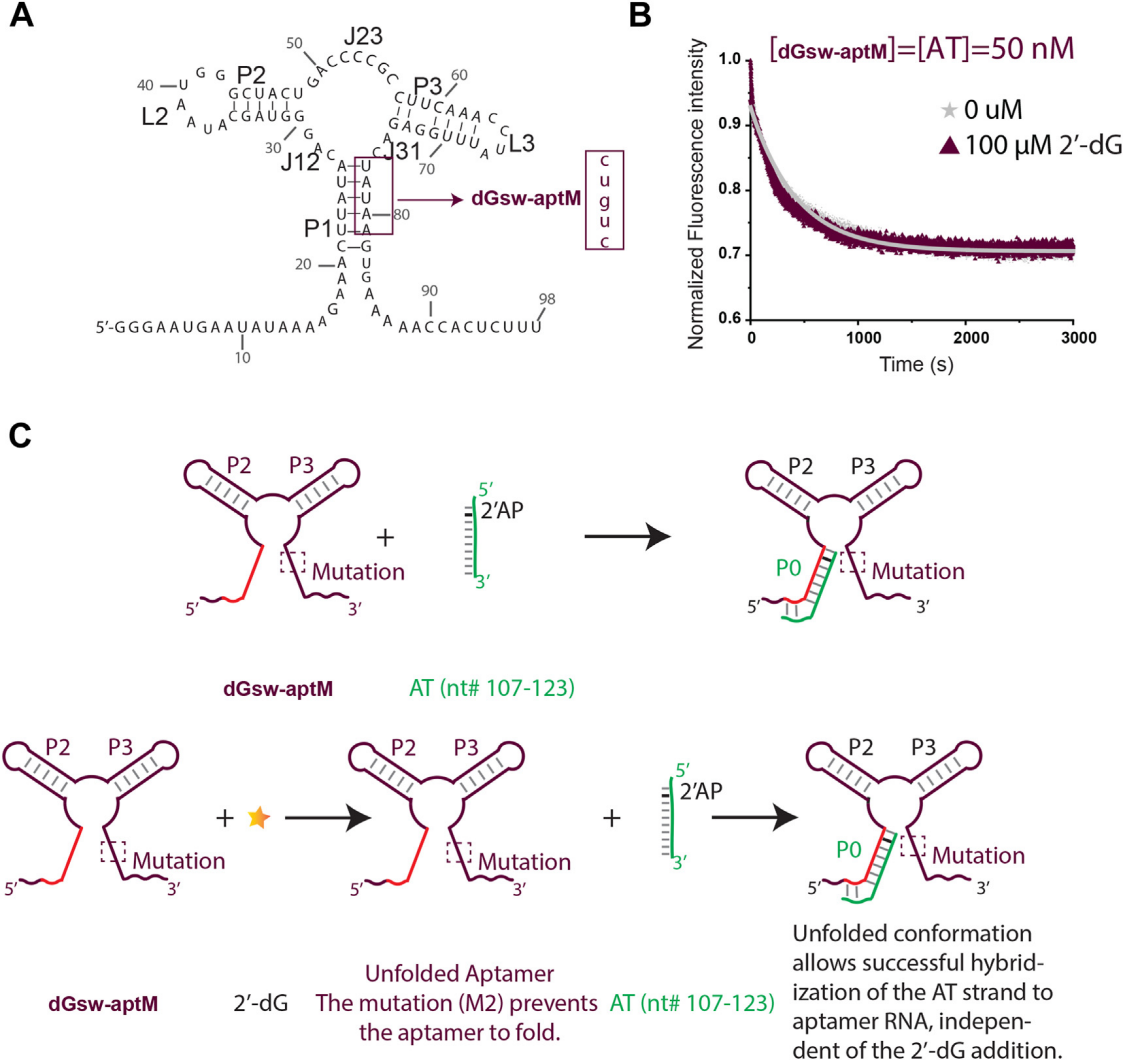
**Dissociation of the P1 helix stabilizes the anti-termination helix**. *A*, the mutated construct (dGsw-aptM) used for the kinetic switching assay. Mutated residues and their replacements are highlighted in the boxed area. *B*, 50 nM of each dGsw-aptM RNA and 2-aminopurine-labeled AT strand were rapidly mixed in the absence and presence of 100 μM of 2′-dG. *C*, scheme for the kinetic switching process. In the ligand-free state, 2-Aminopurine-labelled AT strand anneals with the aptamer domain, forming the AT helix and quenching 2-AP fluorescence. The mutation abrogates P1 helix formation, favoring AT-strand hybridization.

To validate that the decrease in quenching rate is due to competition between a 2′-dG-stablized P1 helix and the anti-terminator helix, we designed a mutant (dGsw-aptM) where P1-helix formation is abrogated (Fig. 5*A*). We then measured fluorescence quenching from dGsw–aptM interactions with the 2-AP-labeled AT-strand in the presence or absence of 2′-dG (Fig. 5, *B* and *C*). The extent of 2-AP quenching for the mutant is comparable to WT quenching in a ligand-free state, further confirming weak association of the P1 helix in the apo state. Interestingly, there is no appreciable change in the 2-AP fluorescence intensity as a function of 2′-dG addition (Fig. 5*B*), indicating that 2′-dG cannot salvage or restore P1 structure in the mutant. These results suggest that dissociation of the P1 helix is essential for both the AT-helix formation and achieving the functional ON-state conformation. Additionally, 2′-dG partially pushes the equilibrium toward the OFF-state conformation by stabilizing the P1 helix. The formation-dissociation equilibrium of P1 is influenced by the ligand concentrations, leading to functionalized ON/OFF states.

## Discussion

RNA molecules are key intermediates in transferring genetic information, a concept famously described in the "central dogma" of molecular biology. Despite significant progress in recent decades, unraveling the intricate folding patterns of messenger RNA (mRNA), especially within noncoding regions, remains challenging. Here, we map the structural details, down to the nucleotide level, of the 5′ untranslated regions of mRNA encoding the β-subunit of ribonucleotide reductase in *M*. *florum* (20). This research offers insight into the evolution of folding pathways and pinpoints specific stages during transcription where regulatory decisions are made.

**Figure 6:**
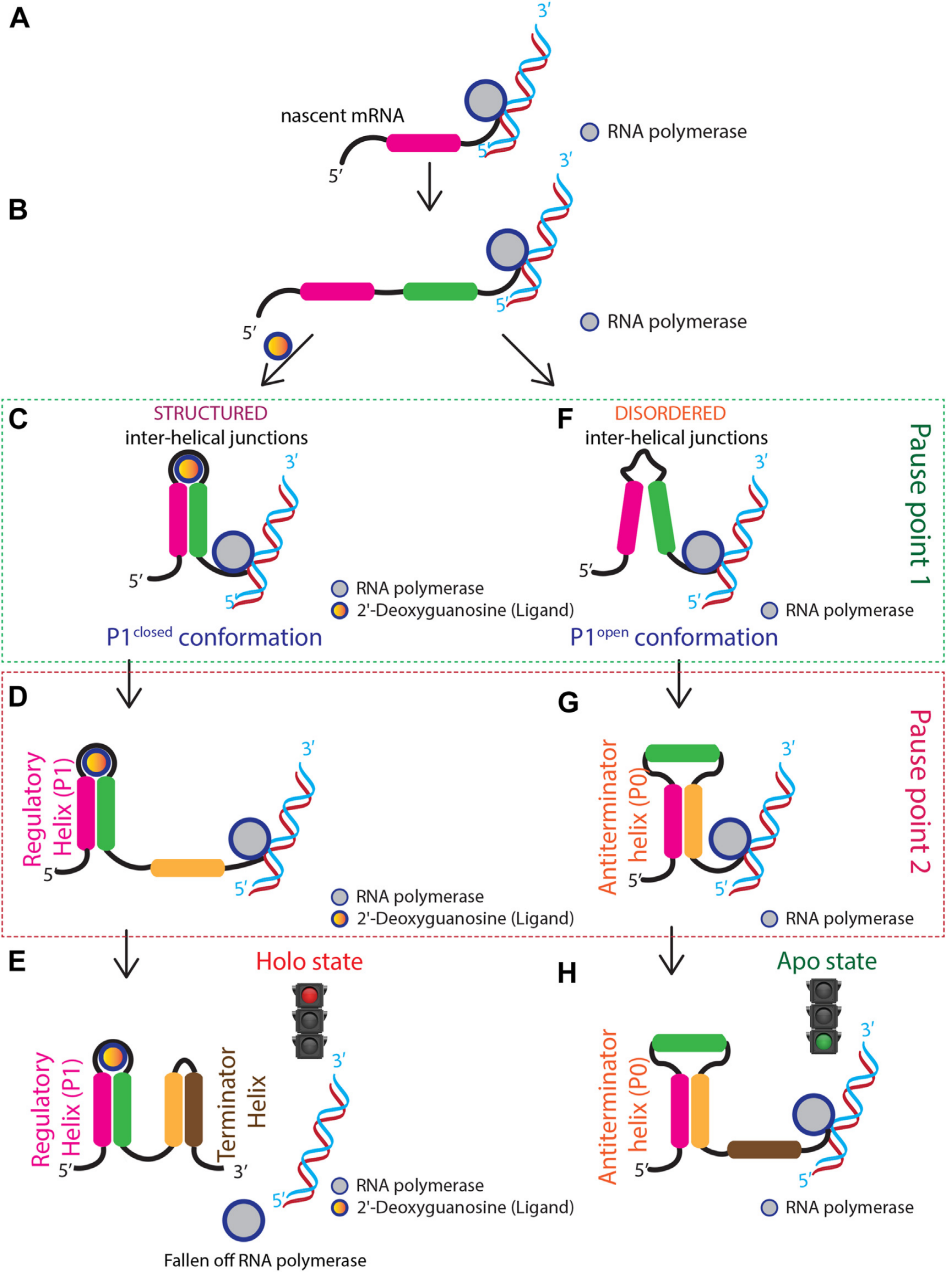
**The outcome for genetic regulation by dGsw is determined by a combination of transcriptional intermediate state and ligand concentrations**. *A*, the RNA polymerase initiates transcription of the nascent mRNA element from corresponding DNA. *B*, as soon as the 5′ strand (*purple*) and 3′-strand (*green*) of the P1 helix is transcribed, the transcription is paused. At this moment, the dGsw functional state can be described in two different scenarios. In Case 1, when 2′-dG is abundant. *C*, rapid binding of 2′-dG triggers a global folding event within the aptamer domain. Consequently, the P1 helix undergoes a transition to a more compact conformation. *D*, the RNA polymerase pausing at nucleotides 93 to 103 (*orange*) allows sufficient time for the aptamer to complete the folding process. *E*, following the folding process, RNA polymerase transcribes the terminator helix, terminating transcription after residue U143 (*brown*). This conformation describes the genetic OFF state. In Case 2, under conditions of 2′-dG depletion. *F*, the P1 helix of the aptamer domain is less rigidly folded. The pausing of RNA polymerase at nucleotides 93 to 103 does not induce structural modulation under these conditions. *G*, a pausing of polymerase after residue U118 provides an opportunity for hybridization of the AT-strand (*orange*) to P1a (*purple*), forming the antiterminator (P0) helix. *H*, the AT helix heralds the genetic ON state, permitting transcription of the downstream ribonucleotide reductase gene.

The functionality of the 2′-dG-sensing transcriptional regulator of the ribonucleotide reductase gene is contingent upon two critical factors: transcript length and 2′-dG concentration. While the dGsw-fl does not undergo a global conformational change in response to 2′-dG, transcriptional intermediates such as dGsw-int adopt distinct conformations that are critical for the regulatory function. This dGsw-int provides a mechanistic explanation for how the riboswitch fine-tunes gene expression during transcription. Previous investigations by Helmling *et al*. identified pivotal transcriptional pause sites near residues 93 to 103 and residue U118 (28, 30). Employing constructs derived from these pause points, we delineate the branching process governing dGsw activity, as summarized in Figure 6. The dGsw-int construct (nt 1–123) corresponds to a transcriptional intermediate validated by NMR profiling and *in vitro* transcription assays (28, 30). This region includes a U-rich pause site (nt 118–123) that delays RNAP elongation, allowing the aptamer domain and antiterminator helix to fold co-transcriptionally (30, 41). While the 3′-terminal nucleotides may remain partially occluded within the RNAP exit channel (∼20–25 nt), the 5′ regions required for P1 and antiterminator formation are fully accessible. This intermediate represents a metastable ON-state ensemble critical for regulation, as shown by its ligand-independent structural features (28, 30) and synchronization with bacterial transcription rates (30).

Briefly, the dGsw functional state can be described in two different scenarios. In Case 1, when 2′-dG is abundant, rapid binding of 2′-dG triggers a global folding event within the aptamer domain. Consequently, the P1 helix undergoes a transition to a more compact conformation. The RNA polymerase pausing at nucleotides 93 to 103 allows ample time for the aptamer to complete the folding process. Previous studies have determined that the ligand binding to purine aptamer domains is indeed quite rapid, with rates ranging from ∼10^4^–10^5^ M^−1^ s^−1^ (18, 27). Following the folding process, RNA polymerase transcribes the terminator helix, terminating transcription after residue U143. Our SHAPE probing data demonstrates that the simultaneous presence of 2′-dG and Mg^2+^ drives the formation of a folded aptamer domain and a structured terminator helix, typical of the genetic OFF state.

In Case 2, under conditions of 2′-dG depletion, the P1 helix of the aptamer domain is less rigidly folded. The pausing of RNA polymerase at nucleotides 93 to 103 does not induce structural modulation under these conditions. Instead, a pause site after residue U118 provides an opportunity for hybridization of the AT-strand to P1a, forming the AT helix. The AT helix heralds the genetic ON state, permitting transcription of the downstream ribonucleotide reductase gene.

Our results provide a detailed structural framework for understanding the function of dGsw, laying a foundation for elucidating structure-function relationships in transcriptional riboswitches. This research is essential to unravel how bacteria, such as *M*. *florum*, maintain cellular deoxyribonucleotide homeostasis. It sheds light on how prokaryotes may have shaped the gene regulation process *via* transcriptional intermediates.

Previous work highlighted the necessity for ligand binding to occur before synthesizing the expression platform. (13, 18, 42). Single-molecule force spectroscopy showed that the aptamer domain of the adenine riboswitch folds co-transcriptionally in a hierarchical manner (29). These findings and our proposed model (Fig. 6) are consistent, as both conclude that intermediates mediate the transition between the P1 and AT helices, a process that governs transcription rates.

The structural analysis using static (SHAPE) and dynamic (fluorescence) experiments performed under near-thermodynamic equilibrium conditions indicates that a kinetic component is required to adopt the antiterminator fold. Without the second pause point after the AT-strand (Fig. 6) (30), there would likely be a coexistence of ligand-dependent and ligand-independent functional OFF-states, hindering the transcription of downstream genes. Notably, this transcriptional model is also observed for the FMN riboswitch, where the pausing of the bacterial polymerase is required for FMN binding and subsequent folding into the OFF-state conformation (17).

This work has important implications for understanding mRNA transcriptional regulation and its connection to cellular processes such as purine metabolism in bacteria. By elucidating the structural profiles of prokaryotic noncoding RNAs interacting with cellular metabolites, we gain insights into the mechanisms of small-molecule sensing, with potential applications in drug discovery and RNA-targeted therapeutics. For example, our work suggests that biotechnological application of transcription-regulating riboswitches in heterologous organisms may require synchronization between the pausing of RNA polymerase and the conformational switching of the riboswitch. With their modularity and ligand specificity, riboswitches hold promise for applications in inducible gene expression systems, offering targeted control over gene expression in response to specific ligands (43).

## Experimental procedure

### PCR and *in vitro* transcription of RNA

Different constructs of dGsw RNAs were transcribed from a synthetic double-stranded DNA template by run-off transcription (44). The DNA templates corresponding to different 2′-dG constructs were purchased as synthetic gblock DNA from Integrated DNA Technologies (Table S1) and were subsequently amplified by PCR. The corresponding DNA template was transcribed using an AmpliScribe T7 High Yield Transcription Kit (LGC Biosearch Technologies) following the manufacturer's protocol. The resulting RNAs were precipitated by adding three volumes of absolute ethanol and one volume of 7 M ammonium acetate, followed by centrifugation at 4°C at 16000g for 30 min. The concentration of each RNA construct was calculated from the absorbance at 260 nm using the molar extinction coefficient of each RNA, calculated using the server https://www.fechem.uzh.ch/MT/links/ext.html. RNA quality was verified using a denaturing 8% polyacrylamide gel electrophoresis gel.

### 2′-hydroxyl acylation of 2′-dG riboswitch by N-Methylisatoic anhydride probing and primer extension

Purified RNA was resuspended in nuclease-free water, and 30 pmole was thermally denatured by heating at 95°C for 5 min, followed by snap cooling on ice for 2 min. Each RNA construct was refolded at 20°C in a buffered medium (50 mM Na-HEPES, 100 mM KCl, pH 8.0) in the presence or absence of 5 mM of MgCl_2_ and 20 μM 2′-dG. To ensure complete saturation of the binding site of riboswitch, we used a concentration of 2′-dG that is 250-fold higher than the K_D_ (5). This approach guarantees that the riboswitch is fully bound to 2′-dG during our probing experiments. Following incubation at 20°C for 30 min, 1-Methyl-7-nitroisatoic anhydride (1M7) in anhydrous DMSO was added to RNA samples to a final concentration of 3 mM. Samples were incubated at 25°C for 10 min, then RNAs were precipitated by adding three volumes of 100% cold ethanol, 1/10th volume of 3 M sodium acetate, and 25 μg glycogen (Thermo Fisher Scientific; Waltham, MA). RNA was pelleted by centrifugation at 4°C at 16000g for 30 min. In parallel, control samples are prepared using neat DMSO instead of 1M7.

RNA samples modified with 1M7 were resuspended in 7.75 μl of nuclease-free water. RNAs were then mixed with 1 μl of 1.5 μM (1.5 pmole) 5′-Alexa-488 labeled Unirev primer, followed by successive incubations at 65°C for 1 min, 45°C for 5 min, and on ice for 1 min (45). The primer extension mix was supplemented with 500 μM of each dNTP, 10 mM DTT, 1X SSIII FS buffer, and 200 U/μl of SuperScript III Reverse Transcriptase (Invitrogen). A 15 μl primer extension reaction was initiated by incubating the mixture at 55°C for 1.25 h. Adenosine and guanosine sequencing reactions were performed identically on thermally denatured unmodified RNA. For sequencing reactions (Fig. S1), the primer extension mix was supplemented with 333 μM ddTTP and ddCTP (Cytiva), respectively. Primer extension reactions were desalted using P-6 micro-biospin columns (BioRad).

RNA samples were diluted in deionized formamide (Hi-Di Formamide, Thermo Fisher Scientific) in a 1:20 ratio (by volume) and heated to 95°C for 3 to 4 min. The samples were electrokinetically injected (30 s at 6 kV) onto an ABI Prism 3100 Avant quad-capillary instrument. A fluorescence electropherogram was then collected at 14 kV. Each electropherogram was processed as previously described (22, 34, 35, 36, 37, 38, 39). Briefly, data were aligned and integrated using in-house software to simultaneously fit multiple Gaussian peaks to the traces. The integrated area for individual nucleotides corresponding to the blank sample (RNA treated with neat DMSO) was then subtracted from SHAPE data (1M7 treated). To normalize the data, 10% of the residues with the highest reactivity values are considered outliers and were temporarily excluded from the analysis. From the remaining data, 10% of highly reactive nucleotides were averaged to calculate a normalization factor. The entire profile, including the previously excluded outliers, was then normalized by that factor (33, 46). On the normalized scale, reactive nucleotides were defined as those with reactivities higher than 0.6 and unreactive nucleotides were those with reactivities below 0.3.

All SHAPE experiments were performed in triplicate, and the data presented in the manuscript are the best of these replicates.

### 2-Aminopurine switching assay

The aptamer domain of dGsw (dGsw-apt) and the mutant (dGsw-aptM) were transcribed and purified as described above. The purified RNA (50 nM, 100 μl) was denatured at 95°C for 5 min followed by snap cooling on ice for 2 min. Individual RNAs were folded in a buffered medium (50 mM Na-HEPES, 100 mM KCl, 5 mM Mg^2+^, pH 8.0). The synthetic RNA Anti-terminator (AT) strand was procured from Integrated DNA Technologies (Coralville, IA) containing a 2-amino purine analog in the following sequence: 5′-A(2-AP)GUUUCUUUUUAUGUC-3’. The refolding/folding of the AT strand was performed as described above. Fluorometric data were collected on a FluoroMax Plus-C fluorometer (Horiba Jobin Yvon) using 310 nm excitation and 372 nm emission wavelengths, with slit widths of 5 nm. RNA aptamers were diluted to 50 nM and incubated at 25°C for 20 min in the presence or absence of 100 μM 2′-dG. The solution of the folded aptamer RNA was then rapidly mixed with an equal volume (100 μl each) of the AT strand in a 400 μl cuvette while data were collected. The observed rate constants (k_obs_) in the presence or absence of ligands were calculated by fitting initial (40s) kinetic data in a single exponential decay function. Reported K_obs_ uncertainty values were obtained from either the error in the fits, or the standard deviation in values from three technical replicates, whichever was larger.

## Data availability

The data that support the findings are available from the corresponding author on request.

## Supporting information

This article contains supporting information (32, 47).

## Conflict of interest

The authors declare that they have no conflicts of interest with the contents of this article.

## Supporting information

Supportin Information
