# Supplementary material for: Regulatory helix plays a key role in genetic ON–OFF switching for the 2′-deoxyguanosine-sensing mRNA element
Source: J Biol Chem. 2025 May 22;301(7):110282. doi: 10.1016/j.jbc.2025.110282 (PMC12268651; doi:10.1016/j.jbc.2025.110282)
Supplement: Supportin Information [file mmc1.pdf]

Supplementary Information

Regulatory helix plays a key role in genetic ON-OFF switching for the 2'-deoxyguanosine sensing mRNA element

Susmit Narayan Chaudhury[1], Nathan Edward Jespersen[1], Scott P Hennelly[1] and Karissa Y Sanbonmatsu[1,2]*

[1]*Theoretical Biology and Biophysics, Los Alamos National Laboratory, Los Alamos, NM 87545, United States*

[2]*New Mexico Consortium, Los Alamos, NM 87544, United States*

Supplementary Table 1

Template DNA or RNA oligo sequences used in this article

RED: T7 site,
BLACK: Target RNA sequence,
PURPLE: Site of mutations,
GREEN: 3'structure cassette

DNA oligos	Sequence (5'-3)'	Purpose	Procured from
T7 forward primer	GAATTCTAATACGACTCACTATA	Forward primer for PCR amplification	Integrated DNA Technologies (Coralville, IA)
UniRev	ATCGAACCGAACCGAAGCCCAATTT	Reverse primer for PCR amplification.	
**Name of corresponding RNA**	**Sequence (5'-3)'**	**Purpose**	
dGsw-apt	GAATTCTAATACGACTCACTATAGGG<br>AATGAATATAAAAGAAACTTATACA<br>GGGTAGCATAATGGGCTACTGACCCC<br>GCCTTCAAACCTATTTGGAGACTATA<br>AGTGAAAAACCACTCTTTCCGATCCG<br>CTTCGGCGGATCCAAATTGGGCTTCG<br>GTTCGGTTCGATAA	In vitro transcription	
dGsw-int	GAATTCTAATACGACTCACTATAGGG<br>AATGAATATAAAAGAAACTTATACA<br>GGGTAGCATAATGGGCTACTGACCCC<br>GCCTTCAAACCTATTTGGAGACTATA<br>AGTGAAAAACCACTCTTTAATTATTA<br>AAGTTTCTTTTTATGTCCCGATCCGCT<br>TCGGCGGATCCAAATTGGGCTTCGGT<br>TCGGTTCGATAA		
dGsw-fl	GAATTCTAATACGACTCACTATAGGG<br>AATGAATATAAAAGAAACTTATACA<br>GGGTAGCATAATGGGCTACTGACCCC<br>GCCTTCAAACCTATTTGGAGACTATA<br>AGTGAAAAACCACTCTTTAATTATTA<br>AAGTTTCTTTTTATGTCCAAAAGACA<br>AGAAGAAACTTTTTTCCGATCCGCTT<br>CGGCGGATCCAAATTGGGCTTCGGTT<br>CGGTTCGATAA		

dGsw-aptM<br>(mutation at nt77-81)	GAATTCTAATACGACTCACTATAGGG<br>AATGAATATAAAAGAAACTTATACA<br>GGGTAGCATAATGGGCTACTGACCCC<br>GCCTTCAAACCTATTTGGAGACCTGT<br>CGTGAAAAACCACTCTTTCCGATCCG<br>CTTCGGCGGATCCAAATTGGGCTTCG<br>GTTCGGTTCGATAA		
**RNA oligos**	**Sequence (5'-3)'**		
AT<br>(nt#107-123)	A(2-AP)GUUUCUUUUUAUGUC	**Purpose**	
		Fluorometric<br>"Two-piece"<br>assay	

Supplementary Methods

1 Native PolyAcrylamide Gel Electrophoresis (PAGE)

To assess the conformational states of the riboswitch, we performed 8% native TBM (0.5X TBE, 2mM $MgCl_2$) gel electrophoresis. dGsw-fl and dGsw-int RNAs were diluted to appropriate concentrations in 50mM HEPES buffer, pH 8.0, heat-denatured at 95°C for 5 minutes, and then snap-cooled on ice for 2 minutes. Following the addition of 5mM $MgCl_2$, RNAs were incubated for 30 minutes at 37°C. We analyzed the RNA conformations using 8% native PAGE supplemented with 1.1 mM $MgCl_2$. The gel was then run in TBM buffer at 110 V and 22°C for 90 minutes. The gel was stained with Ethidium Bromide and visualized under UV light.

2 RNAProbe Analysis

For independent validation of our SHAPE data analysis, we used the RNAProbe web server(1). We uploaded our SHAPE reactivity and blank data files to the server. The normalization protocol by RNAProbe excluded the first 10% of highest reactivity values as outliers, then calculated the average of the next 10% of highly reactive nucleotides to calculate a normalization factor. The entire profile, including previously excluded outliers, was then normalized by this factor.

3 RNAstructure R-sample Analysis

To deconvolute potential structural ensembles, we performed R-sample analysis using the RNAstructure(2) algorithm. We analyzed our RT-stop data for the following conditions: dGsw-fl+2'-dG, dGsw-fl, dGsw-int+2'-dG, and dGsw-int. The R-sample algorithm generated multiple potential structural conformations for each condition, and the associated free energies of these conformations were calculated.

Supplementary Figure 1

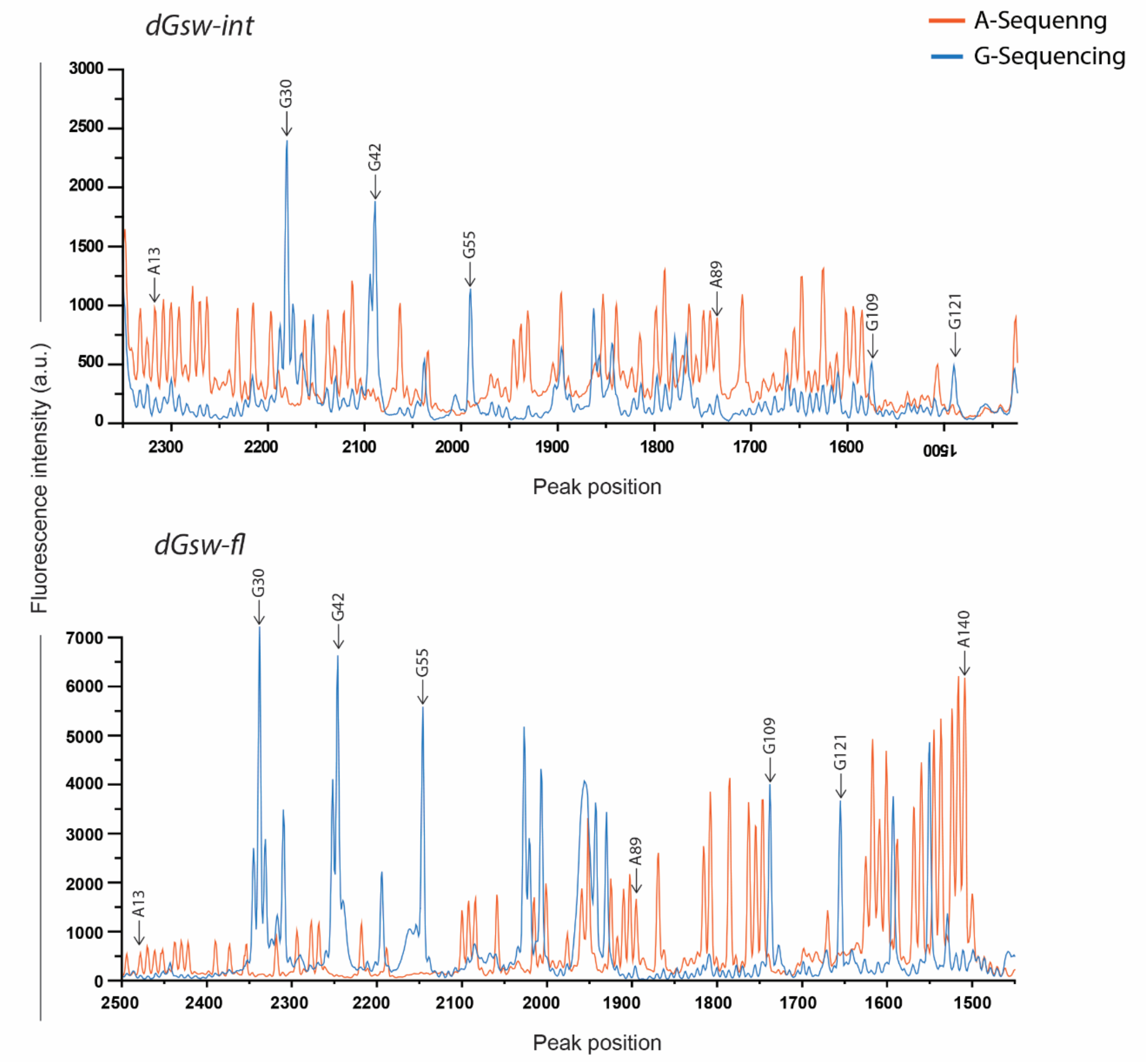

Supplementary Figure 1: A and G sequencing electropherogram for *dGsw-fl* and *dGsw-int* transcripts. Sequencing reactions are performed on thermally denatured unmodified RNA. For sequencing reactions, the primer extension mix is supplemented with 333 uM ddTTP and ddCTP, respectively.

Supplementary Figure 2

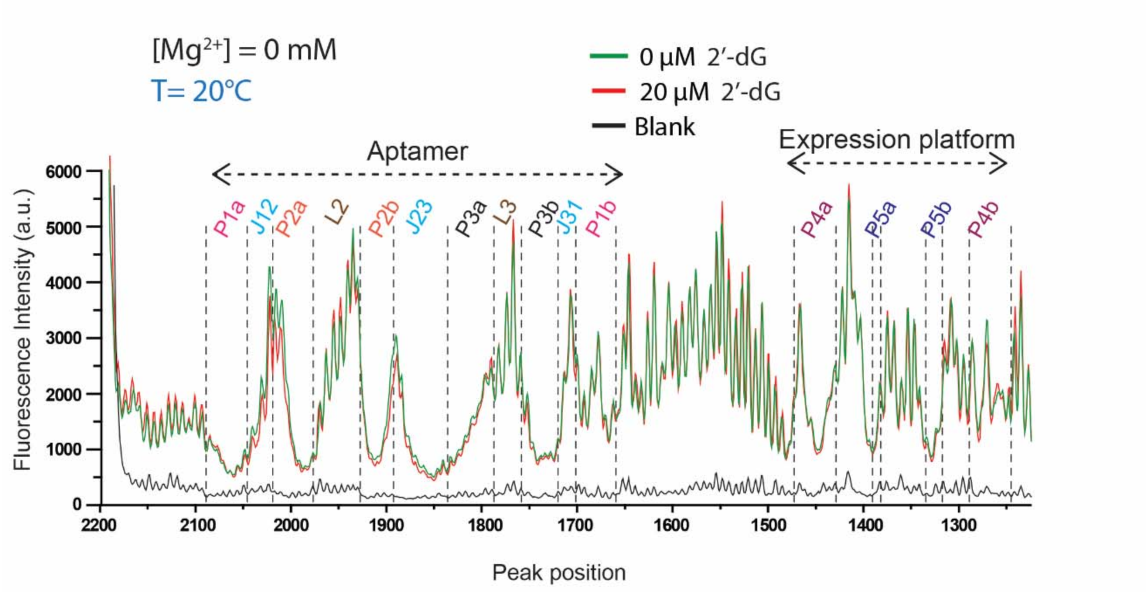

Supplementary Figure 2: Mg^{2+} ion is required to drive the *dGsw-fl* riboswitch into a competent conformation for ligand binding. The SHAPE probing experiments are conducted in the absence of Mg^{2+} at 20°C. Nucleotides from each region are indicated using vertical dotted lines. Overlaid traces show no 2'dG (GREEN), 20 μM 2'dG (RED), and respective blank (BLACK) data. Representative electropherogram in the absence of Mg^{2+}. In the absence of Mg^{2+}, 2'dG (ligand) does not affect the conformational modulation of the *mlf*-2'-dG-fl riboswitch. There is no substantial change in the pattern of the electropherogram in response to the ligand. Interestingly, in absence of Mg^{2+}, the expression platform is intrinsically structured.

Supplementary Figure 3

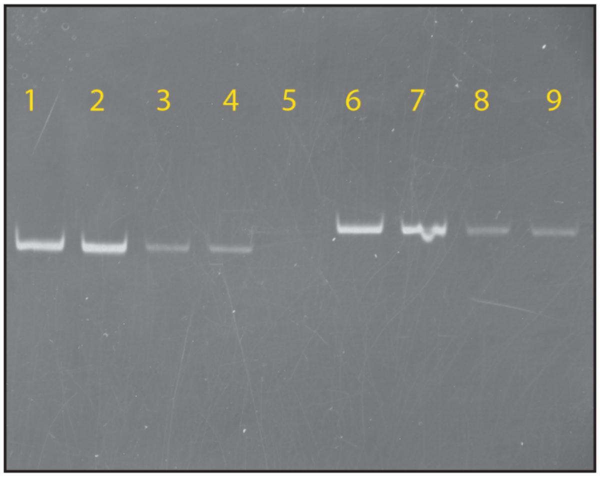

Supplementary Figure 3: 8% native PAGE analysis of dGsw-int and dGsw-fl RNA conformations in the presence and absence of 2'-dG. Lane assignments: (1) 150 ng dGsw-int; (2) 150 ng dGsw-int + 100 µM 2'-dG; (3) 50 ng dGsw-int; (4) 50 ng dGsw-int + 100 µM 2'-dG; (5) Empty; (6) 150 ng dGsw-fl; (7) 150 ng dGsw-fl + 100 µM 2'-dG; (8) 50 ng dGsw-fl; (9) 50 ng dGsw-fl + 100 µM 2'-dG. Native gel electrophoresis reveals that both dGsw-int and dGsw-fl RNAs predominantly exist in a single conformational state. No significant mobility shift was observed for either RNA construct upon addition of 100 µM 2'-dG, suggesting that any binding-induced structural changes are localized and do not alter the overall RNA conformation substantially. This contrasts with our SHAPE structural analysis, which indicated distinct conformational differences between the apo and holo states of dGsw-int RNA. The discrepancy likely arises because the ligand-induced conformational changes involve local tertiary rearrangements that preserve the overall molecular dimensions and surface charge distribution of the RNA, making these structural transitions undetectable by native PAGE, which primarily separates molecules based on dimension/size.

Supplementary Figure 4

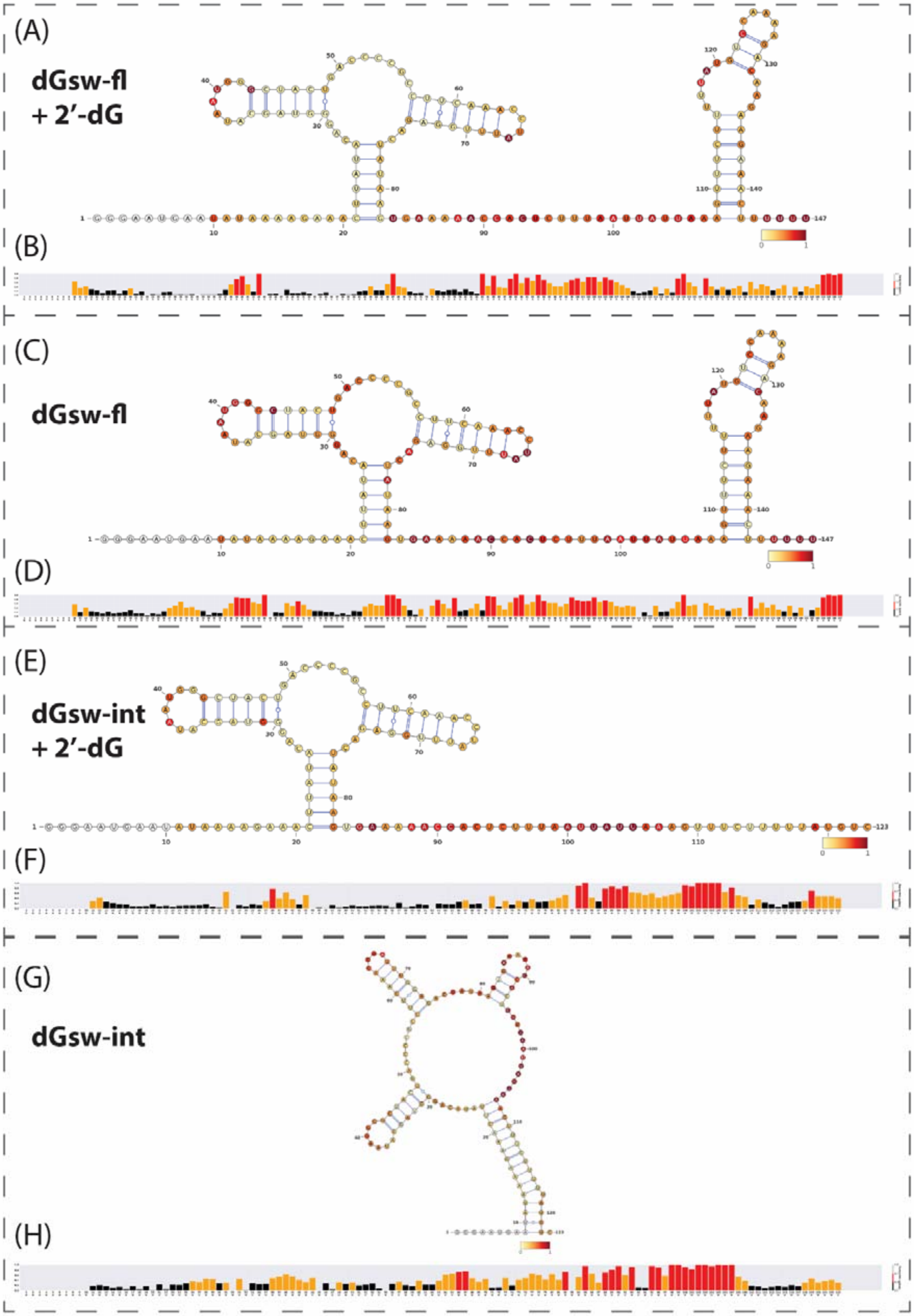

Supplementary Figure 4: SHAPE analysis reveals distinct nucleotide dynamics of dGsw-fl and dGsw-int with and without ligand binding. (A) The secondary structure model of dGsw-fl in the presence of 2'-dG ligand with SHAPE reactivity data was mapped onto the structure. Nucleotides are color-coded according to their SHAPE reactivity as indicated by the color scale. **(B)** Corresponding bar plot representation of SHAPE reactivity values for dGsw-fl + 2'-dG as a function of nucleotide position. **(C)** Secondary structure model of dGsw-fl without ligand with mapped SHAPE reactivities. **(D)** Bar plot showing SHAPE reactivity values for ligand-free dGsw-fl. **(E)** Secondary structure model of the intermediate length transcript (dGsw-int) in the presence of 2'-dG ligand with mapped SHAPE reactivities. **(F)** Bar plot showing SHAPE reactivity profile for dGsw-int + 2'-dG. **(G)** Secondary structure model of ligand-free dGsw-int with SHAPE reactivities. **(H)** Bar plot showing SHAPE reactivity values for ligand-free dGsw-int. The SHAPE data were analyzed using the RNAProbe web server(1). The normalization protocol by RNAProbe excluded the first 10% of highest reactivity values as outliers, then calculated the average of the next 10% of highly reactive nucleotides to calculate a normalization factor. The entire profile, including previously excluded outliers, was then normalized by this factor. The color code distinguishes reactive (and thus unconstrained) nucleotides: red indicates high SHAPE reactivity (>0.7), orange shows medium reactivity (0.3-0.7), and white represents low reactivity (<0.3). Grey circles indicate unanalyzable nucleotides. Comparative analysis of nucleotide-specific conformational flexibility between the full-length construct and dGsw-int upon 2'-dG introduction elucidates the structural rearrangements induced by 2'-dG binding interactions

Supplementary Figure 5

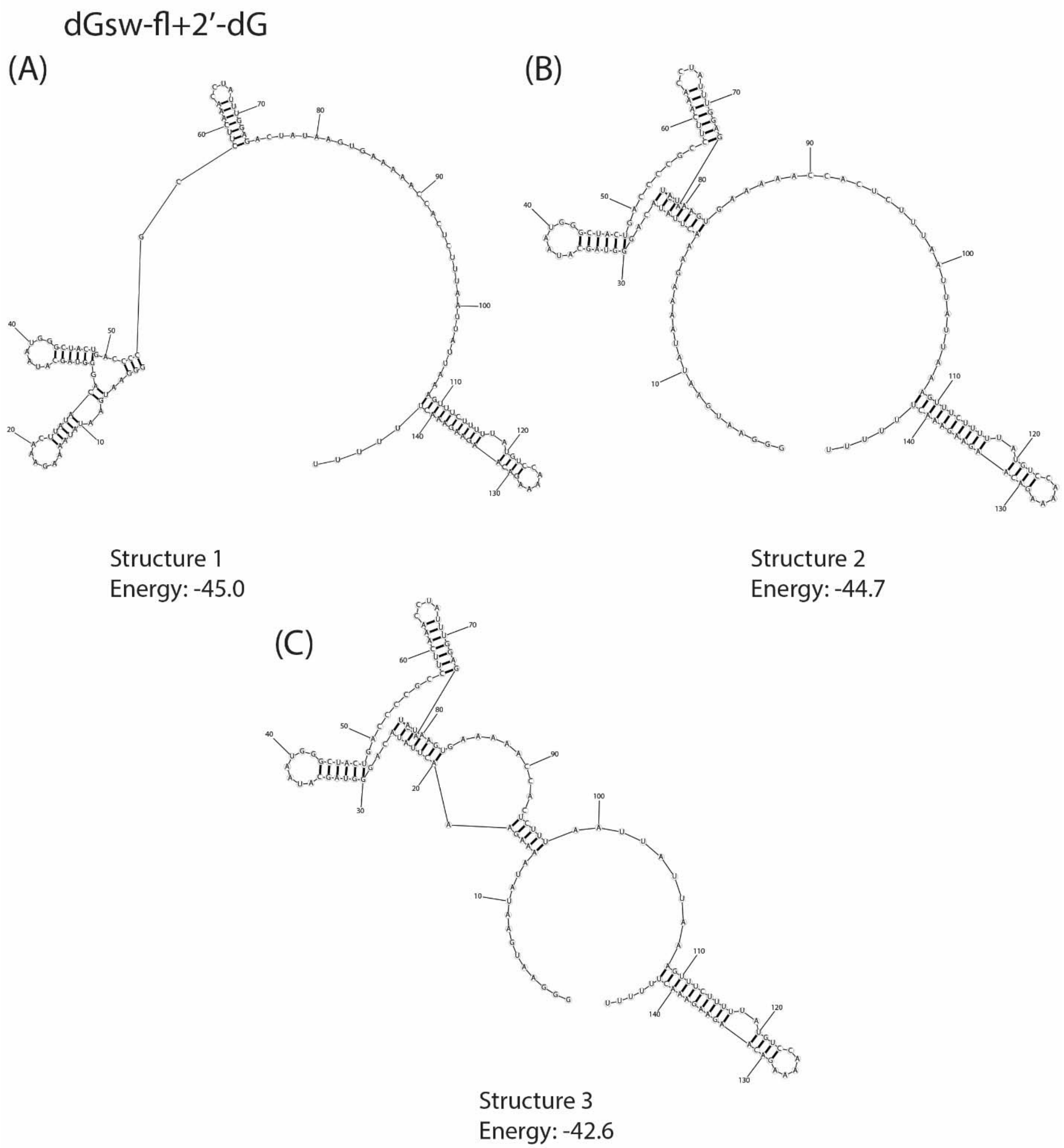

Supplementary Figure 5: R-sample predicted structures for dGsw-fl+2'-dG. (**A-C**) Three potential conformations of the dGsw-fl+2'-dG construct with calculated free energies (kcal/mol) of -45.0, -44.7, and -42.6 kcal/mol, respectively. Structure 1 represents the most energetically favorable conformation.

Supplementary Figure 6

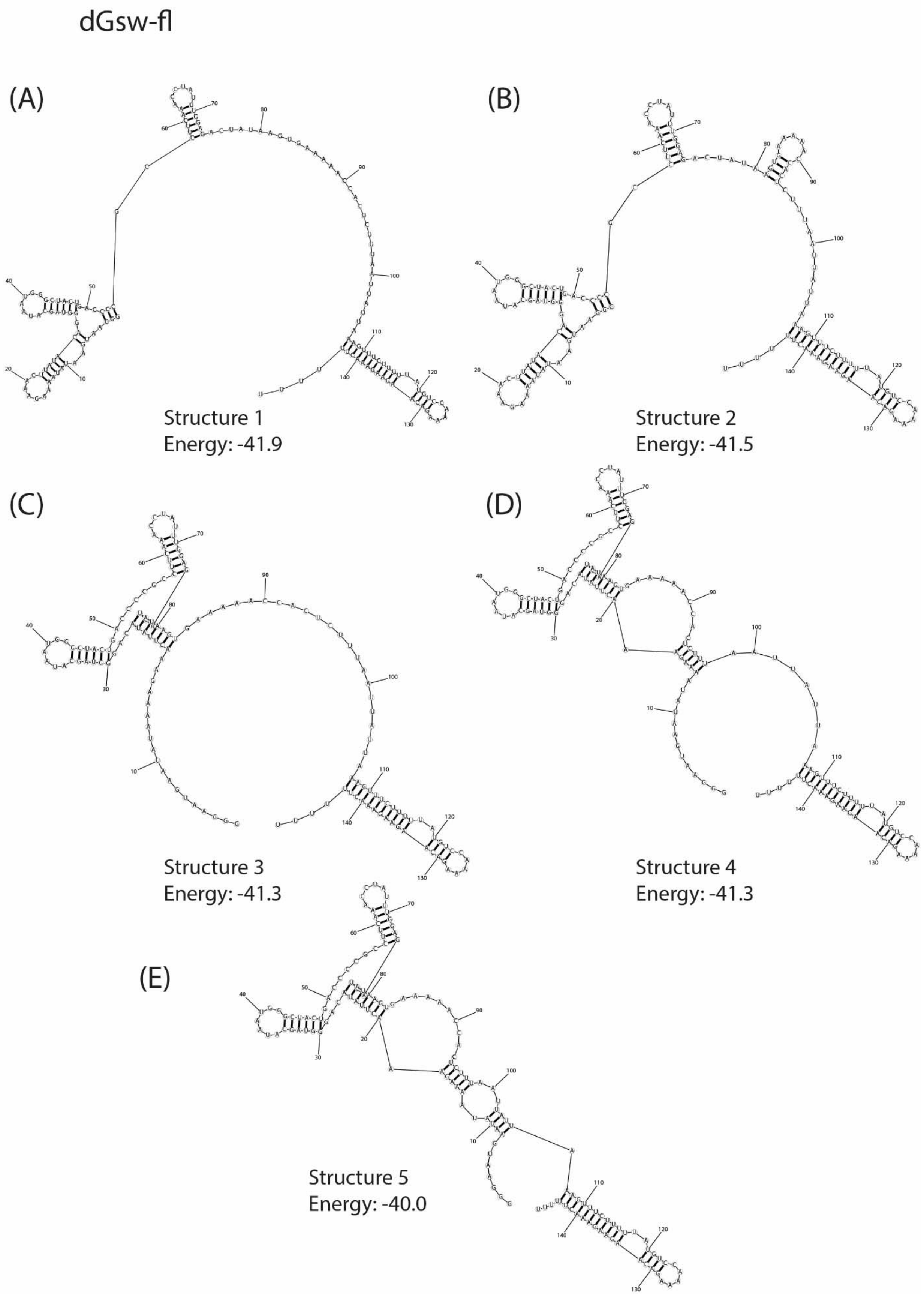

Supplementary Figure 6: R-sample predicted structures for dGsw-fl. (**A-E**) Five potential conformations of the dGsw-fl construct with calculated free energies (kcal/mol) ranging from -41.9 to -40.0 kcal/mol. The energy differences between these structures are relatively small, suggesting conformational flexibility around a dominant structure.

Supplementary Figure 7

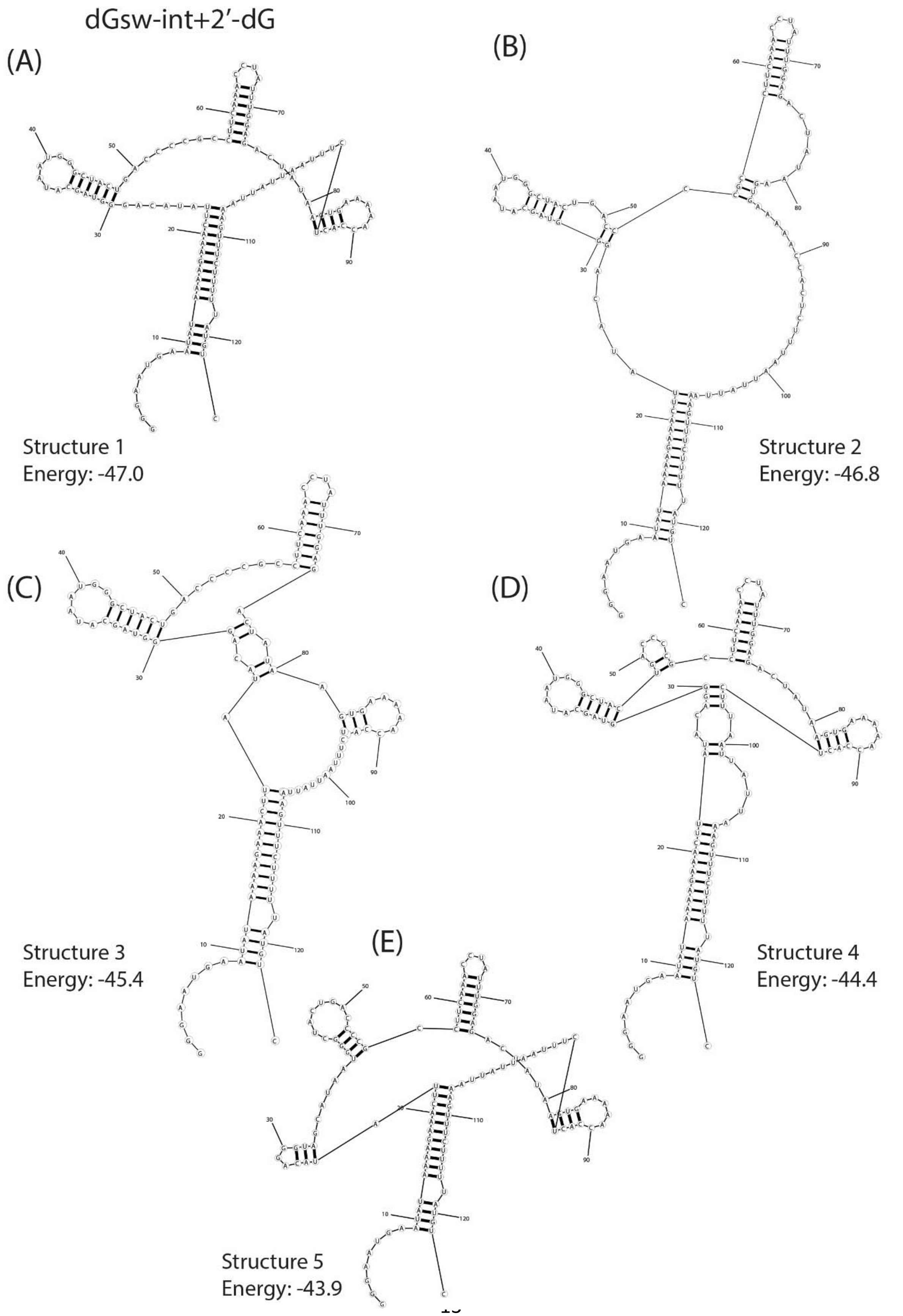

Supplementary Figure 7: R-sample predicted structures for dGsw-int+2'-dG. (**A-E**) Five potential conformations of the dGsw-int+2'-dG construct with calculated free energies (kcal/mol) ranging from -47.0 to -43.9 kcal/mol. Structure 1 shows the most energetically favorable conformation at -47.0 kcal/mol.

Supplementary Figure 8

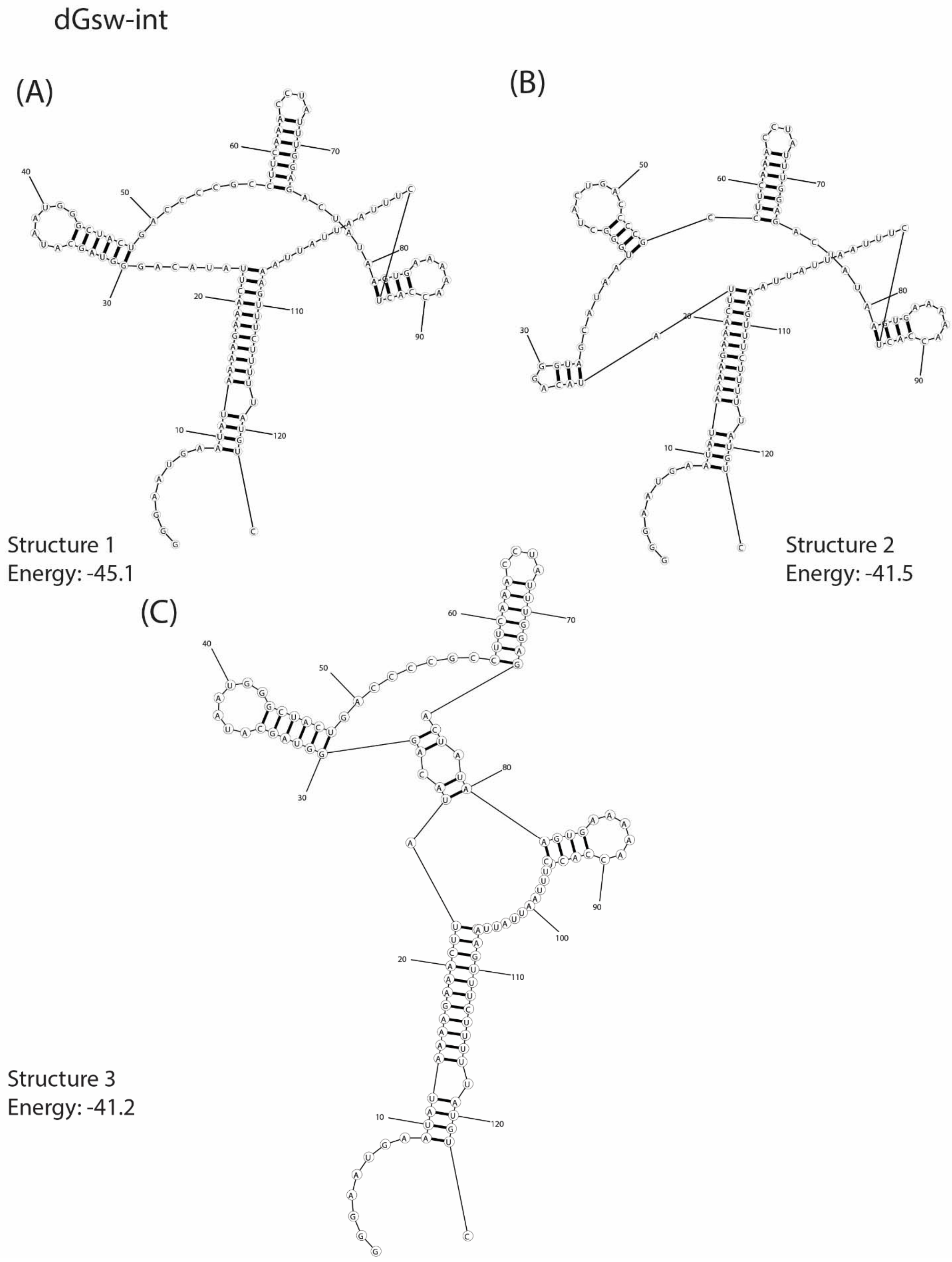

Supplementary Figure 8: R-sample predicted structures for dGsw-int. (A-C) Three potential conformations of the dGsw-int construct with calculated free energies (kcal/mol) of -45.1, -41.5, and -41.2 kcal/mol, respectively. The significant energy difference between Structure 1 and the other conformations suggests it is the dominant structure under these conditions.

References

1. Wirecki, T. K., Merdas, K., Bernat, A., Boniecki, M. J., Bujnicki, J. M., and Stefaniak, F. (2021) RNAProbe: A web server for normalization and analysis of RNA structure probing data. *Nucleic Acids Res.* **48**, W292–W299
2. Deigan, K. E., Li, T. W., Mathews, D. H., and Weeks, K. M. (2009) Accurate SHAPE-directed RNA structure determination. *Proc. Natl. Acad. Sci. U. S. A.* **106**, 97–102
